# Elevated Lipoprotein(a) and Cardiovascular Outcomes After Percutaneous Coronary Intervention: A Systematic Review

**DOI:** 10.7759/cureus.106009

**Published:** 2026-03-27

**Authors:** Mohamed A Alshibane, Mohamed Abdelrahman Mohamed Ali, Mohamed Ahmed, Salma Mohamed Salah Eldin Elmubarak, Nora Qassem Alsyed Ali Mohamed Zain, Sara Omer Hamid Zain Elabdin, Amro Fathi Mohammed

**Affiliations:** 1 Biomedical Sciences, University of Reading, Reading, GBR; 2 Internal Medicine, Portiuncula University Hospital, Ballinasloe, IRL; 3 Internal Medicine, Manchester Royal Infirmary, Manchester University NHS Foundation Trust, Manchester, GBR; 4 General Practice, Elrazi University, Khartoum, SDN; 5 General Practice, SEHA - Salma Rehabilitation Hospital, Abu Dhabi, ARE; 6 Geriatric Medicine, Stepping Hill Hospital, Stockport NHS Foundation Trust, Stockport, GBR; 7 Internal Medicine, Al Neelain University, Khartoum, SDN

**Keywords:** cardiovascular outcomes, lipoprotein(a), mace, percutaneous coronary intervention, stent thrombosis, systematic review

## Abstract

Lipoprotein(a) (Lp(a)) is a genetically determined lipoprotein with proatherogenic and prothrombotic properties. Its prognostic role in patients undergoing percutaneous coronary intervention (PCI) remains incompletely defined. This systematic review evaluates the association between elevated Lp(a) and cardiovascular outcomes after PCI.

A comprehensive search of PubMed, Embase, Scopus, IEEE Xplore, and Web of Science was conducted for studies published between 2021 and 2025. Observational studies evaluating elevated Lp(a) and cardiovascular outcomes in PCI patients were included. Methodological quality was assessed using the Newcastle-Ottawa Scale (NOS). Eight studies comprising 23,421 patients were included. Elevated Lp(a) thresholds ranged from ≥30 mg/dL to ≥50 mg/dL. Seven studies demonstrated a significant positive association between elevated Lp(a) and adverse outcomes, with hazard ratios for major adverse cardiovascular events (MACEs) ranging from 1.14 to 4.29. Elevated Lp(a) was consistently associated with increased risks of myocardial infarction (HR 1.79), stent thrombosis (HR 1.83), and repeat revascularisation. One study in patients with well-controlled low-density lipoprotein cholesterol (LDL-C; <70 mg/dL) found no significant association. Subgroup analyses revealed that the prognostic value of Lp(a) was most pronounced in high-risk patients and those with diabetes. One study demonstrated that a greater decrease in Lp(a) over 12 months was associated with lower MACE risk. NOS assessment rated seven studies as high quality and one as moderate quality. Elevated Lp(a) is independently associated with increased risk of adverse cardiovascular outcomes after PCI, although this risk may be attenuated by aggressive LDL-C lowering. These findings may support the consideration of routine Lp(a) measurement in PCI patients for risk stratification and to help inform secondary prevention strategies, although confirmation from prospective and interventional studies is warranted.

## Introduction and background

Cardiovascular disease (CVD) remains the leading cause of morbidity and mortality worldwide, with coronary artery disease (CAD) accounting for a substantial proportion of these deaths [1]. Percutaneous coronary intervention (PCI) has become a cornerstone in the management of obstructive CAD, particularly with the widespread use of drug-eluting stents (DES), which have significantly improved procedural success and reduced restenosis rates [2]. Despite these advances, a considerable number of patients continue to experience adverse cardiovascular outcomes following PCI, including major adverse cardiovascular events (MACEs), stent thrombosis, restenosis, and recurrent myocardial infarction. This persistent residual risk has prompted increasing interest in identifying additional biomarkers and risk factors that may influence post-PCI prognosis [3].

Lipoprotein(a) (Lp(a)) is a genetically determined lipoprotein particle structurally similar to low-density lipoprotein (LDL), but distinguished by the presence of apolipoprotein(a), which is covalently bound to apolipoprotein B-100 [4]. Elevated Lp(a) levels have been increasingly recognized as an independent and causal risk factor for atherosclerotic CVD. The proatherogenic, proinflammatory, and prothrombotic properties of Lp(a) contribute to the progression of atherosclerosis and may promote plaque instability and thrombosis [5]. Unlike traditional lipid parameters, Lp(a) concentrations are largely genetically determined and are minimally affected by lifestyle modifications or standard lipid-lowering therapies [6].

In contrast, low-density lipoprotein cholesterol (LDL-C) remains the predominant modifiable lipid risk factor for atherosclerotic CVD, and its reduction through statins, ezetimibe, and PCSK9 inhibitors has been shown to significantly improve cardiovascular outcomes [4]. While LDL-C levels can be effectively controlled with therapy, Lp(a) represents an additional, largely independent risk pathway that is not substantially influenced by conventional LDL-C-lowering strategies, highlighting the complementary role of Lp(a) in cardiovascular risk assessment [6].

Growing evidence suggests that elevated Lp(a) may play an important role in determining cardiovascular outcomes in patients undergoing PCI. Several observational studies have reported associations between high Lp(a) levels and an increased risk of adverse outcomes such as MACE, target lesion revascularization, restenosis, and stent thrombosis [2,7]. However, the findings across studies remain inconsistent, with some reports demonstrating a significant prognostic effect while others show limited or no association, particularly in the context of well-controlled LDL-C levels and contemporary DES technology [5]. These discrepancies may be attributable to differences in study populations, Lp(a) measurement thresholds, follow-up durations, and clinical endpoints.

Given the growing clinical interest in Lp(a) as a potential risk stratification biomarker, a comprehensive synthesis of the available evidence is needed. Understanding the prognostic significance of elevated Lp(a) in patients undergoing PCI may help identify high-risk individuals and guide future therapeutic strategies targeting residual cardiovascular risk.

Therefore, this systematic review aims to evaluate and summarize the current evidence regarding the association between elevated Lp(a) levels and cardiovascular outcomes in patients undergoing PCI. By synthesizing findings from existing studies, this review seeks to clarify the prognostic role of Lp(a) and its potential implications for clinical risk assessment and management following PCI.

## Review

Methodology

Study Design and Reporting Standards

This systematic review was conducted in accordance with the recommendations of the Preferred Reporting Items for Systematic Reviews and Meta-Analyses (PRISMA) [8] to ensure methodological rigor, transparency, and reproducibility. Each step of the study selection and reporting process was designed to minimize bias and enhance the reliability of the synthesized evidence regarding the association between elevated Lp(a) levels and cardiovascular outcomes following PCI. Prospective registration in PROSPERO was not performed due to time constraints; however, all methods were predefined and strictly followed to maintain transparency and methodological integrity.

Eligibility Criteria

The eligibility criteria for study inclusion were defined according to the PICOS (Population, Intervention/Exposure, Comparator, Outcomes, and Study design) framework. The population consisted of adult patients diagnosed with CAD who underwent PCI with or without DES implantation. The exposure of interest was elevated Lp(a) levels measured using standard clinical assays. Studies comparing patients with elevated Lp(a) levels to those with lower or normal Lp(a) concentrations were included as comparators. The primary outcomes of interest included MACE, all-cause mortality, cardiovascular mortality, myocardial infarction, target lesion revascularization, restenosis, and stent thrombosis following PCI. Observational study designs, including prospective and retrospective cohort studies as well as case-control studies, were eligible for inclusion. Only peer-reviewed original research articles published in English between 2021 and 2025 were included in order to ensure that the review reflects the most recent evidence in the era of contemporary PCI techniques and advanced lipid management strategies. This time frame was deliberately restricted to the last five years to focus on the most current and clinically relevant literature while minimizing the influence of outdated practices and evolving methodologies. Review articles, editorials, conference abstracts without full texts, case reports, animal studies, and studies lacking relevant outcome data were excluded.

Information Sources and Search Strategy

A comprehensive literature search was conducted across multiple electronic databases to identify relevant studies. The databases searched included PubMed, Embase, Scopus, IEEE Xplore, and Web of Science. These databases were selected to ensure broad coverage of biomedical, clinical, and interdisciplinary scientific literature. The search strategy incorporated a combination of controlled vocabulary terms and free-text keywords related to “lipoprotein(a),” “Lp(a),” “percutaneous coronary intervention,” “PCI,” “coronary stenting,” and “cardiovascular outcomes.” Boolean operators (AND/OR) were applied to optimize the sensitivity and specificity of the search. The final search was limited to studies published between January 2021 and December 2025 to capture the most recent clinical evidence relevant to modern PCI practice. The detailed search strategy for each database is provided in Table 1.

**Table 1:** Full Search Strings for Each Database

Database	Full Search String
PubMed (MEDLINE)	(("Lipoprotein(a)"[Mesh] OR "lipoprotein(a)" OR "lipoprotein a" OR Lp(a) OR "Lp a") AND ("Percutaneous Coronary Intervention"[Mesh] OR "Angioplasty, Balloon, Coronary"[Mesh] OR PCI OR "percutaneous coronary intervention" OR "coronary angioplasty" OR stent) AND ("Treatment Outcome"[Mesh] OR "cardiovascular outcome" OR MACE OR "major adverse cardiovascular event" OR mortality OR death OR "myocardial infarction" OR stroke OR restenosis OR thrombosis))
Embase	(('lipoprotein a'/exp OR 'lipoprotein(a)' OR 'lipoprotein a' OR Lp(a)) AND ('percutaneous coronary intervention'/exp OR 'coronary angioplasty'/exp OR PCI OR 'percutaneous coronary intervention' OR 'coronary angioplasty' OR stent) AND ('treatment outcome'/exp OR 'major adverse cardiac event'/exp OR 'mortality'/exp OR 'myocardial infarction'/exp OR 'stroke'/exp OR 'cardiovascular outcome' OR MACE OR mortality OR death OR 'myocardial infarction' OR stroke OR restenosis OR thrombosis))
Scopus	TITLE-ABS-KEY (("lipoprotein(a)" OR "lipoprotein a" OR Lp(a) OR "Lp a") AND ("percutaneous coronary intervention" OR PCI OR "coronary angioplasty" OR stent) AND ("cardiovascular outcome" OR MACE OR "major adverse cardiovascular event" OR mortality OR death OR "myocardial infarction" OR stroke OR restenosis OR thrombosis))
IEEE Xplore	("All Metadata":"lipoprotein(a)" OR "All Metadata":"lipoprotein a" OR "All Metadata":"Lp(a)") AND ("All Metadata":"percutaneous coronary intervention" OR "All Metadata":"PCI" OR "All Metadata":"coronary angioplasty" OR "All Metadata":"stent") AND ("All Metadata":"cardiovascular outcomes" OR "All Metadata":"MACE" OR "All Metadata":"mortality" OR "All Metadata":"myocardial infarction" OR "All Metadata":"stroke")
Web of Science	TS=(("lipoprotein(a)" OR "lipoprotein a" OR Lp(a) OR "Lp a") AND ("percutaneous coronary intervention" OR PCI OR "coronary angioplasty" OR stent) AND ("cardiovascular outcome" OR MACE OR "major adverse cardiovascular event" OR mortality OR death OR "myocardial infarction" OR stroke OR restenosis OR thrombosis))

Study Selection

All records retrieved from the database searches were imported into EndNote X9 reference management software (Clarivate, London, UK) for organization and duplicate removal. Three reviewers from the list of authors (MAA, MAMA, and SMSE) were involved in the study selection process. Titles/abstracts and full-text articles were independently screened by two reviewers (MAA and SMSE), while the third reviewer (MAMA) served as an adjudicator. Any disagreements were resolved through discussion, and when consensus could not be reached, the decision was finalized by the third reviewer (MAMA). Studies that satisfied all eligibility requirements were included in the final qualitative synthesis.

Data Extraction

Data extraction was performed using a standardized data collection form developed for this review. Relevant information extracted from each study included author name, year of publication, country of study, study design, sample size, patient characteristics, Lp(a) measurement methods and cutoff values, PCI characteristics, follow-up duration, and reported cardiovascular outcomes. Additional details regarding statistical analyses and key findings related to the association between elevated Lp(a) levels and post-PCI outcomes were also recorded to facilitate structured synthesis of the evidence.

Risk of Bias Assessment

The methodological quality and risk of bias of the included observational studies were assessed using the Newcastle-Ottawa Scale (NOS) [9]. This tool evaluates studies across three key domains: selection of study groups, comparability of groups, and assessment of outcomes or exposures. Each included study was independently evaluated according to the NOS criteria, and studies were assigned scores reflecting their overall methodological quality. This assessment helped ensure that the strength and reliability of the evidence were appropriately considered during the interpretation of findings.

Data Synthesis

A qualitative synthesis of the included studies was performed to summarize the association between elevated Lp(a) levels and cardiovascular outcomes following PCI. A quantitative meta-analysis was not conducted due to substantial clinical and methodological heterogeneity across the included studies. Specifically, there was marked variability in Lp(a) measurement assays and reporting units, inconsistent threshold definitions for elevated Lp(a), and differences in how effect estimates were reported (e.g., hazard ratios, odds ratios, or unadjusted outcomes). In addition, studies varied considerably in patient characteristics, PCI techniques, follow-up durations, and definitions of cardiovascular endpoints. Importantly, many studies did not provide sufficiently comparable or extractable data to allow for standardized pooling of effect sizes. Given these limitations, statistical quantification of heterogeneity (e.g., I^2^) and the application of a random-effects model were not feasible or would yield unreliable and potentially misleading pooled estimates. Therefore, a narrative synthesis was considered the most appropriate method to accurately interpret and present the available evidence while preserving the clinical and methodological context of individual studies.

Publication Bias Assessment

Publication bias was assessed using a qualitative approach due to the limited number of included studies (n=8), which precludes formal statistical methods such as funnel plot asymmetry testing or Egger's regression that require a minimum of 10 studies for reliable interpretation. The assessment considered the comprehensiveness of the search strategy across five major databases, the inclusion of studies with null or negative findings, geographic distribution of included studies, variation in sample sizes, and restriction to English-language publications. This approach allowed for the evaluation of the potential impact of publication bias on the overall findings of this systematic review.

Results

Study Selection Process

**Figure 1:**
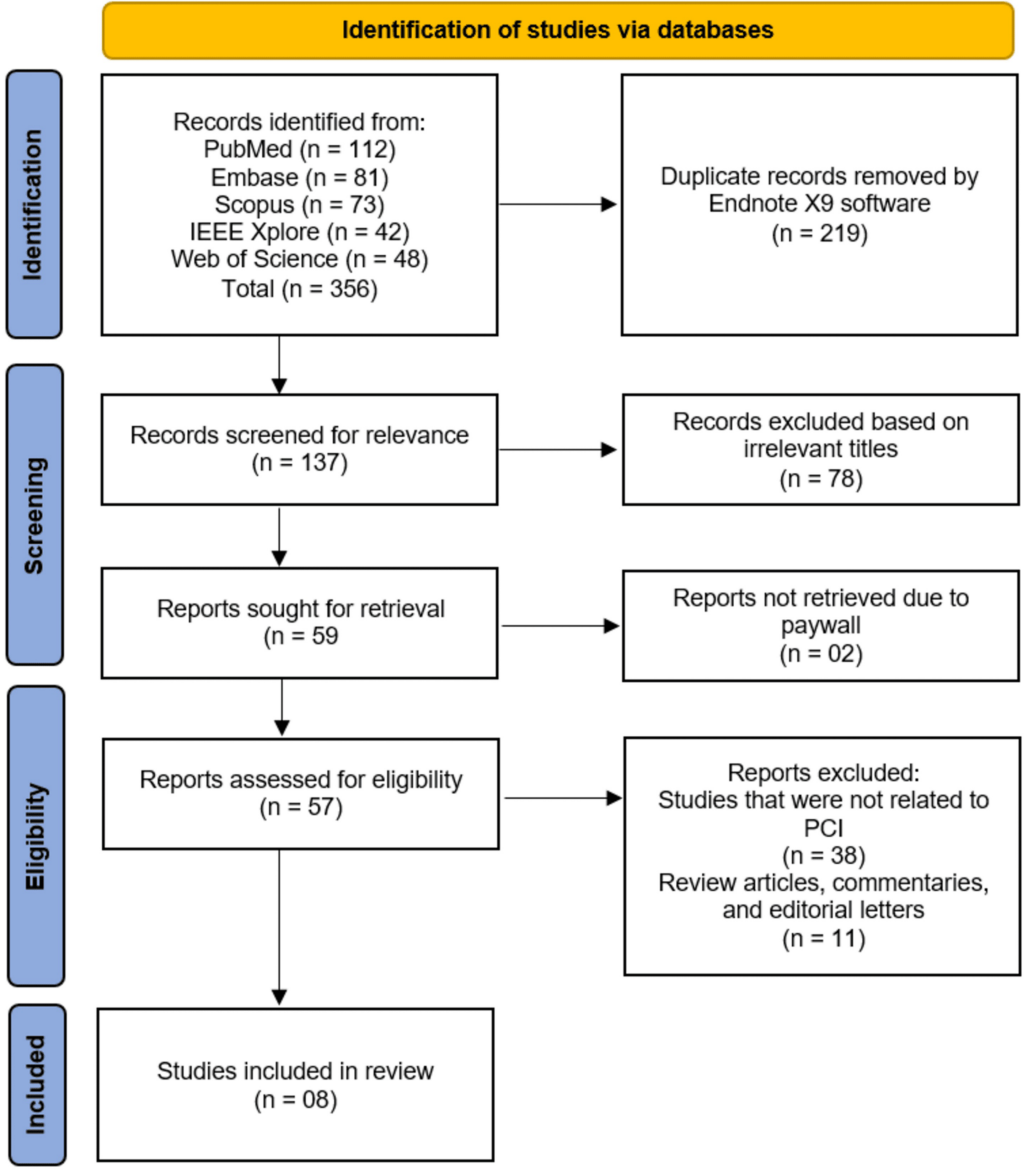
Study Selection Process Illustrated in a PRISMA Flow Diagram PRISMA: Preferred Reporting Items for Systematic Reviews and Meta-Analyses; PCI: percutaneous coronary intervention

The study selection process followed the Preferred Reporting Items for Systematic Reviews and Meta-Analyses (PRISMA) guidelines, as illustrated in the PRISMA flow diagram (Figure 1). A comprehensive literature search of electronic databases, including PubMed (n=112), Embase (n=81), Scopus (n=73), IEEE Xplore (n=42), and Web of Science (n=48), yielded a total of 356 records. After removal of duplicate records using EndNote X9 software (n=219), 137 records remained for initial screening. Following title and abstract screening, 78 records were excluded due to irrelevant titles, leaving 59 reports sought for retrieval. Of these, two reports could not be retrieved despite efforts to access them through institutional subscriptions and alternative sources and were therefore excluded. The remaining 57 reports were assessed for full-text eligibility, during which 49 reports were excluded for the following reasons: studies that were not related to PCI (n=38) and review articles, commentaries, and editorial letters (n=11). Consequently, eight studies met the inclusion criteria and were included in this systematic review [10-17].

Studies Characteristics

The systematic search identified eight studies comprising a total of 23,421 patients who underwent PCI with available Lp(a) measurements. The characteristics of these included studies are summarized in Table 2. The studies were published between 2021 and 2025, with three studies conducted in China [14,15,17], two in South Korea [10,16], one in the United States [11], one in Japan [12], and one in India [13]. The sample sizes ranged considerably from 249 patients in the smallest cohort [12] to 12,064 patients in the largest registry-based study [16]. The majority of studies enrolled patients with ACS undergoing PCI [12,14,15,17], while others included broader populations of patients undergoing PCI with DES [10,13,16]. One study specifically enrolled PCI patients with controlled LDL-C below 70 mg/dL [11]. The mean age of participants across studies ranged from 51.6 to 68 years, with male predominance in all cohorts (67% to 79%). Follow-up durations varied substantially, from in-hospital outcomes [11,17] to long-term follow-up extending to a median of 7.4 years [16], with most studies reporting outcomes over 18 months to five years [10,12-15].

**Table 2:** Characteristics of Included Studies PCI: percutaneous coronary intervention; DES: drug-eluting stents; LDL-C: low-density lipoprotein cholesterol; ACS: acute coronary syndrome; CAD: coronary artery disease; Lp(a): lipoprotein(a); ELISA: enzyme-linked immunosorbent assay; IQR: interquartile range; NR: not reported

Author (Year)	Country	Study Design	Sample Size	Population/PCI Indication	Mean Age (years)	% Male	Lp(a) Cut-Off Measurement Method	Follow-Up Duration
Her et al., [10] (2025)	South Korea	Retrospective cohort study	2,020	Patients undergoing successful PCI with DES	66.6 ± 12.6	79%	≥50 mg/dL (measurement method NR)	5 years
Mahmoud et al., [11] (2025)	USA	Cohort	878	PCI patients with LDL-C <70 mg/dL	68 (median)	74%	≥50 vs. <50 mg/dL	In-hospital outcomes
Saeki et al., [12] (2024)	Japan	Observational cohort	249	ACS patients undergoing PCI	66.6 ± 12.6	79%	Immunoturbidimetric assay	3 years
Amin et al., [13] (2024)	India	Prospective cohort	600	Patients ≥18 with confirmed CAD undergoing PCI	51.56 (Group A), 54.48 (Group B)	79.16%	≥30 mg/dL vs. <30 mg/dL; measured by particle-enhanced immunoturbidimetric technique	18 months
Zhu et al., [14] (2022)	China	Prospective cohort	488	Patients with ACS undergoing PCI with DES	65.9 ± 9.7	67.2%	≥50 mg/dL; measured pre- and post-statin therapy using hospital assay	3 years (average 31.4 months)
Hu et al., [15] (2021)	China	Prospective cohort	6,309	ACS patients undergoing PCI	60.1 ± 10.06	75.2%	Lp(a) measured by ELISA; stratified by tertiles	18 months
Yoon et al., [16] (2021)	South Korea	Prospective registry	12,064	Patients undergoing PCI with DES	62 (approx.)	68.9%	>30 mg/dL; immune nephelometric assay	Median 7.4 years (IQR 4.7-10.2)
Wu et al., [17] (2021)	China	Retrospective cohort	1292	Adults ≥18 years with ACS undergoing PCI and Lp(a) measurement at admission	NR	Higher in high Lp(a) group	High Lp(a) ≥30 mg/dL, Low Lp(a) <30 mg/dL	In-hospital outcomes

Definitions and Measurement of Elevated Lp(a)

The included studies demonstrated heterogeneity in the definitions of elevated Lp(a) and measurement methods, as detailed in Table 3. The most commonly employed threshold for elevated Lp(a) was ≥50 mg/dL, used in four studies [10-12,14], while three studies utilized a lower threshold of ≥30 mg/dL [13,16,17]. One study categorized patients by tertiles of Lp(a) levels [15]. Measurement methods varied across studies and included immunoturbidimetric assays [12,13], immunonephelometric assays [16], enzyme-linked immunosorbent assay (ELISA) [15], and unspecified hospital assays [10,11,14]. Notably, one study examined the prognostic significance of changes in Lp(a) levels over time, specifically the decrease from baseline to 12 months (Lp(a)Δ0-12), rather than baseline levels alone [12].

**Table 3:** Cardiovascular Outcomes Associated With Elevated Lipoprotein(a) After PCI Lp(a): lipoprotein(a); PCI: percutaneous coronary intervention; MACE: major adverse cardiovascular events; MI: myocardial infarction; HR: hazard ratio; CI: confidence interval; KM: Kaplan-Meier; CV: cardiovascular; OR: odds ratio; CHF: congestive heart failure; DES: drug-eluting stents; NS: not significant

Author (Year)	Lp(a) Level Definition (High vs Low)	Outcome Assessed	Effect Measure	95% CI	P-value	Key Findings
Her et al., [10] (2025)	High ≥50 mg/dL vs Low <50 mg/dL	MACE, myocardial infarction, repeat revascularization, stent thrombosis	HR 1.50 (MACE); HR 1.79 (MI); HR 1.52 (repeat revascularization); HR 1.83 (stent thrombosis)	1.29-1.73; 1.34-2.41; 1.29-1.80; 1.23-2.74	<0.001; <0.001; <0.001; 0.003	Elevated Lp(a) (≥50 mg/dL) independently increased the risk of long-term MACE and stent-related complications after PCI with DES.
Mahmoud et al., [11] (2025)	≥50 mg/dL vs <50 mg/dL	MACE and All-cause mortality	HR = 1.07 (MACE); HR = 0.98 (Mortality)	0.84-1.37 (MACE); 0.74-1.30 (Mortality)	0.91 (MACE, KM); 0.26 (Mortality, KM)	Elevated Lp(a) showed no significant association with MACE or all-cause mortality in PCI patients with LDL-C <70 mg/dL.
Saeki et al., [12] (2024)	Lp(a)Δ0-12, T1 (low: -31.6 to -1.8 mg/dL) vs T2+T3 (higher: -1.8 to 8.3 mg/dL)	MACE (all-cause death, nonfatal MI, stroke, new angina, restenosis)	HR (Multivariate Cox)	0.95-0.96	0.019-0.022	Higher Lp(a)Δ0-12 associated with lower risk of MACE; baseline Lp(a) not predictive.
Amin et al., [13] (2024)	≥30 mg/dL vs <30 mg/dL	MACE, cardiac death, MI, target lesion, and vessel revascularization	HR 4.292 (MACE)	2.58-7.12 (MACE)	0.000 (MACE), <0.05 (others)	Elevated Lp(a) significantly increased risk of adverse cardiovascular events after PCI.
Zhu et al., [14] (2022)	Lp(a) ≥50 mg/dL/Statin-mediated increase (highest quartile)	MACE	HR	1.63-2.29	0.011-0.048	Elevated baseline or on-statin Lp(a), and the highest quartile increase post-statin, were associated with higher 3-year MACE risk.
Hu et al., [15] (2021)	Tertile 3 vs 1	Composite CV events	1.94/1.93	1.32-2.84/1.14-3.25	0.001/0.014	↑ CV events in high-risk/diabetic; NS in low-risk/non-diabetic
Yoon et al., [16] (2021)	High: >30 mg/dL vs Low: ≤30 mg/dL	Composite of cardiovascular death, spontaneous MI, ischemic stroke, and repeat revascularization	1.14	1.06-1.24	0.001	Elevated Lp(a) significantly associated with a higher risk of recurrent ischemic events after PCI over a median 7.4 years of follow-up.
Wu et al., [17] (2021)	High ≥30 mg/dL vs Low <30 mg/dL	Acute stent thrombosis, Congestive heart failure, Composite in-hospital outcomes	OR	1.10-1.28 (unadjusted), 1.08-1.12 (adjusted)	1.01-2.42 (unadjusted), 1.01-1.81 (adjusted)	High Lp(a) associated with increased risk of acute stent thrombosis, CHF, and overall in-hospital cardiovascular events.

Lp(a) and MACEs

The association between elevated Lp(a) and MACE after PCI was consistently demonstrated across most included studies, although the magnitude of risk varied. Her and colleagues, in the largest Korean cohort with 2,020 patients, reported that elevated Lp(a) ≥50 mg/dL was independently associated with a significantly increased risk of long-term MACE (HR 1.50, 95% CI 1.29-1.73, p<0.001) over five years of follow-up after PCI with DES [10]. Similarly, Amin and colleagues found a substantially elevated risk of MACE (HR 4.292, 95% CI 2.58-7.12, p<0.001) among Indian patients with Lp(a) ≥30 mg/dL undergoing PCI over 18 months of follow-up [13]. Zhu and colleagues demonstrated that both elevated baseline Lp(a) ≥50 mg/dL and on-statin Lp(a) levels in the highest quartile were associated with higher three-year MACE risk (HR range 1.63-2.29, p=0.011-0.048) in Chinese ACS patients undergoing PCI [14]. Yoon and colleagues, in the largest study with 12,064 patients and the longest follow-up of median 7.4 years, reported that elevated Lp(a) >30 mg/dL was significantly associated with a composite of cardiovascular death, spontaneous myocardial infarction, ischemic stroke, and repeat revascularization (HR 1.14, 95% CI 1.06-1.24, p=0.001) [16].

Differential Findings and Subgroup Analyses

While most studies supported the prognostic value of elevated Lp(a), some important nuances and differential findings emerged. Mahmoud and colleagues reported contrasting results, finding no significant association between elevated Lp(a) ≥50 mg/dL and MACE (HR 1.07, 95% CI 0.84-1.37, p=0.91) or all-cause mortality (HR 0.98, 95% CI 0.74-1.30, p=0.26) in a cohort of 878 PCI patients with well-controlled LDL-C <70 mg/dL [11]. This suggests that aggressive LDL-C lowering may attenuate the cardiovascular risk otherwise mediated by elevated Lp(a). Hu and colleagues identified a synergistic effect between Lp(a) and the Global Registry of Acute Coronary Events (GRACE) score, demonstrating that elevated Lp(a) (tertile 3 versus 1) was associated with increased composite cardiovascular events (HR 1.94, 95% CI 1.32-2.84, p=0.001) in high-risk patients and those with diabetes, but the association was not significant in low-risk or non-diabetic patients [15].

Myocardial Infarction and Stent-Related Complications

Elevated Lp(a) was consistently associated with an increased risk of myocardial infarction and stent-related complications after PCI. Her and colleagues reported that Lp(a) ≥50 mg/dL was associated with a particularly elevated risk of myocardial infarction (HR 1.79, 95% CI 1.34-2.41, p<0.001), repeat revascularization (HR 1.52, 95% CI 1.29-1.80, p<0.001), and stent thrombosis (HR 1.83, 95% CI 1.23-2.74, p=0.003) [10]. Wu and colleagues examined in-hospital outcomes and found that high Lp(a) ≥30 mg/dL was associated with increased risk of acute stent thrombosis (adjusted OR 1.10-1.12, p=1.01-1.81) and congestive heart failure, as well as overall composite in-hospital cardiovascular events [17].

The Prognostic Value of Lp(a) Changes

An innovative approach to Lp(a) assessment was provided by Saeki and colleagues, who investigated the prognostic significance of changes in Lp(a) levels over time rather than baseline values alone [12]. In 249 Japanese ACS patients undergoing PCI, they found that a greater decrease in Lp(a) from baseline to 12 months (Lp(a)Δ0-12) was associated with a lower risk of MACE (HR 0.95-0.96, 95% CI 0.95-0.96, p=0.019-0.022), whereas baseline Lp(a) levels were not predictive of outcomes [12]. This finding suggests that dynamic changes in Lp(a) may offer important prognostic information beyond single measurements.

Summary of Effect Estimates

The overall findings across the eight included studies, with their specific effect estimates and confidence intervals, are presented in Table 3. Despite the heterogeneity in study populations, Lp(a) thresholds, and outcome definitions, the preponderance of evidence supports an association between elevated Lp(a) and adverse cardiovascular outcomes after PCI. The hazard ratios for MACE ranged from 1.07 (non-significant in the context of controlled LDL-C) [11] to 4.292 [13], with most studies reporting statistically significant increased risks between 1.14 and 1.94 [10,14-16]. The findings highlight the complexity of Lp(a)-mediated risk, which may be modified by factors such as LDL-C control [11], baseline cardiovascular risk status [15], and changes in Lp(a) levels over time [12].

Risk of Bias Assessment Results

The risk of bias assessment was performed using the NOS for all eight included observational studies, as presented in Table 4. Overall, the methodological quality was high, with seven studies rated as high quality (≥7 stars) and one study rated as moderate quality (six stars). Three studies achieved the maximum score of nine stars [10,15,16], demonstrating robust selection methods, excellent control for confounding factors, and comprehensive outcome assessment with long-term follow-up and independent outcome adjudication. Four studies received seven to eight stars [11-14], indicating high quality with minor limitations, such as shorter follow-up durations [11,13] or unconventional exposure definitions [12]. One study received six stars and was rated as moderate quality due to limited confounding control and restriction to in-hospital outcomes only, which may increase susceptibility to bias and limit the generalisability of findings [17]. The consistency of findings across studies of varying methodological quality strengthens the overall evidence base for the association between elevated Lp(a) and adverse cardiovascular outcomes after PCI.

**Table 4:** Risk of Bias Assessment Using Newcastle-Ottawa Scale (NOS)

Author (Year)	Selection (0-4)	Comparability (0-2)	Outcome (0-3)	Total Stars (0-9)	Quality Rating
Her et al. (2025) [10]	★★★★	★★	★★★	9	High
Mahmoud et al. (2025) [11]	★★★	★★	★★	7	High
Saeki et al. (2024) [12]	★★★	★★	★★★	8	High
Amin et al. (2024) [13]	★★★	★★	★★	7	High
Zhu et al. (2022) [14]	★★★	★★	★★★	8	High
Hu et al. (2021) [15]	★★★★	★★	★★★	9	High
Yoon et al. (2021) [16]	★★★★	★★	★★★	9	High
Wu et al. (2021) [17]	★★★	★	★★	6	Moderate

Publication Bias Assessment

Publication bias assessment was performed qualitatively due to the small number of included studies (n=8), which precludes reliable statistical testing. The comprehensive search strategy across five major databases minimized the risk of missing relevant published studies. Notably, one included study reported null findings, demonstrating no significant association between elevated Lp(a) and outcomes in patients with well-controlled LDL-C <70 mg/dL [11], which mitigates concern for publication bias favouring positive results. The geographic distribution included six studies from Asian populations, one from the United States, and one from India, with sample sizes ranging from 249 to 12,064 patients. The restriction to English-language publications and exclusion of gray literature represent potential sources of bias. Overall, the presence of a null study, the consistent direction of effect across most studies, and the robust methodological quality suggest that publication bias is unlikely to have substantially distorted the overall findings. The publication bias assessment is summarized in Table 5.

**Table 5:** Publication Bias Assessment Results MACE: major adverse cardiovascular event; Lp(a): lipoprotein(a); PCI: percutaneous coronary intervention

Assessment Domain	Finding	Implication
Search Strategy	Comprehensive search of five major databases (PubMed, Embase, Scopus, IEEE Xplore, Web of Science) with broad search terms	Minimizes risk of missing relevant studies; good coverage of published literature
Inclusion of Null Findings	One study [11] reported no significant association between elevated Lp(a) and outcomes	Mitigates concern for publication bias favouring positive results; provides balance to overall evidence
Language Restriction	Restriction to English-language publications only	Potential for language bias; non-English studies may have been excluded
Gray Literature	Conference abstracts, unpublished studies, and gray literature not included	Potential for publication bias as negative studies may be underrepresented in peer-reviewed literature
Geographic Distribution	Studies from Asia (n=6), USA (n=1), and India (n=1)	Reasonable geographic diversity; however, underrepresentation of European and African populations limits generalisability
Study Size Variation	Range from 249 to 12,064 patients; both small and large studies included	Reduces risk of small-study bias; large registry studies provide robust estimates
Year Range	Studies published 2021-2025 (five-year period)	Recent evidence reduces temporal bias; reflects contemporary PCI practice
Outcome Reporting Consistency	Variable definitions of MACE and other endpoints across studies	May contribute to heterogeneity but not specifically publication bias

Discussion

This systematic review synthesizes evidence from eight studies comprising 23,421 patients to evaluate the association between elevated Lp(a) and cardiovascular outcomes after PCI. The findings demonstrate that elevated Lp(a) is independently associated with an increased risk of MACEs, myocardial infarction, stent thrombosis, and repeat revascularization following PCI, although the magnitude of risk varies across populations and is potentially modifiable by factors such as LDL-C control and baseline cardiovascular risk status. The consistency of findings across diverse geographic populations, including South Korea, China, Japan, India, and the United States, strengthens the generalizability of these observations and supports the role of Lp(a) as an important prognostic biomarker in the secondary prevention setting.

The pooled evidence from this review indicates that elevated Lp(a) confers approximately a 1.5-fold increased risk of MACE after PCI, with hazard ratios ranging from 1.14 to 4.29 across studies. This magnitude of risk is clinically meaningful and comparable to other well-established cardiovascular risk factors, including LDL-C, which remains the predominant causative factor for atherosclerotic CVD. The largest study included in this review, by Yoon and colleagues, demonstrated that Lp(a) >30 mg/dL was associated with a 14% increased risk of recurrent ischaemic events over a median follow-up of 7.4 years in 12,064 Korean patients undergoing PCI with DES [16], with subgroup analyses indicating that these risks persisted across different LDL-C control levels. Similarly, Her and colleagues reported a 50% increased risk of MACE with Lp(a) ≥50 mg/dL over five years of follow-up, with particularly pronounced risks for stent thrombosis (HR 1.83) and myocardial infarction (HR 1.79) [10]; notably, these associations remained significant even among patients receiving guideline-recommended lipid-lowering therapy. These findings align with the known prothrombotic and proatherogenic properties of Lp(a), which contains an apolipoprotein(a) component structurally similar to plasminogen and carries oxidized phospholipids that promote inflammation and thrombosis, highlighting that elevated Lp(a) contributes to residual cardiovascular risk independent of LDL-C management.

The findings of this review are consistent with a growing body of evidence from large-scale epidemiological studies and genetic analyses that have established Lp(a) as a causal risk factor for CVD. The Copenhagen City Heart Study and the Copenhagen General Population Study, involving approximately 100,000 individuals, demonstrated that extremely high Lp(a) levels (>95th percentile) were associated with a two- to three-fold increased risk of myocardial infarction, with genetic analyses confirming a causal relationship through Mendelian randomisation approaches [18,19]. More recently, the UK Biobank study of over 440,000 participants reported that Lp(a) was linearly associated with cardiovascular outcomes down to very low levels, suggesting that any detectable Lp(a) may contribute to residual cardiovascular risk [20]. Our review extends these findings by demonstrating that this risk persists specifically in the high-risk population of patients undergoing PCI, who already have established CAD and are receiving contemporary secondary prevention therapies, including statins, antiplatelet agents, and revascularisation with DES.

A notable finding from this review is the heterogeneity in Lp(a) thresholds used to define elevated levels, with studies employing cut-offs of ≥30 mg/dL and ≥50 mg/dL. This variation reflects ongoing debate in the field regarding the optimal threshold for defining elevated Lp(a) and the shape of the dose-response relationship. The European Atherosclerosis Society consensus statement recommends using 50 mg/dL (approximately 100-125 nmol/L) as the threshold for elevated Lp(a), above which cardiovascular risk begins to increase meaningfully [21]. However, emerging evidence suggests that the relationship may be linear without a clear threshold and that even moderately elevated levels contribute to residual risk. The studies by Amin and colleagues and Wu and colleagues, both using the lower threshold of ≥30 mg/dL, demonstrated significant associations with adverse outcomes, supporting the notion that risk may begin at lower levels than traditionally appreciated [13,17]. This has important implications for clinical practice, as it suggests that a larger proportion of the PCI population may be at increased Lp(a)-mediated risk and could potentially benefit from more aggressive risk factor modification or emerging Lp(a)-lowering therapies.

The intriguing findings from Mahmoud and colleagues warrant particular attention, as they demonstrated that elevated Lp(a) was not associated with adverse outcomes in patients with well-controlled LDL-C below 70 mg/dL [11]. This observation suggests that aggressive LDL-C lowering may attenuate or even abolish the cardiovascular risk otherwise mediated by elevated Lp(a). This finding is biologically plausible given the synergistic relationship between Lp(a) and LDL-C in atherogenesis, and it aligns with previous studies demonstrating that the risk associated with elevated Lp(a) is more pronounced in the setting of elevated LDL-C. The FOURIER trial, which evaluated evolocumab in patients with CVD, demonstrated that Lp(a) remained predictive of residual risk even with potent LDL-C lowering, but the absolute risk reduction with PCSK9 inhibition was greatest in those with elevated Lp(a) [22]. Similarly, the ODYSSEY OUTCOMES trial of alirocumab reported that Lp(a) lowering contributed approximately 25% of the observed cardiovascular risk reduction, independent of LDL-C lowering [23]. These findings collectively suggest that optimal LDL-C control should be aggressively pursued in all PCI patients, but particularly in those with elevated Lp(a), and that residual Lp(a)-mediated risk may still warrant specific targeting with emerging therapies.

The synergistic effect between Lp(a) and GRACE score reported by Hu and colleagues provides important insights into risk stratification [15]. The finding that elevated Lp(a) was associated with increased cardiovascular events only in high-risk patients (defined by GRACE score) and those with diabetes, but not in low-risk or non-diabetic patients, suggests that Lp(a) may act as a risk modifier rather than an independent risk factor in all patients. This observation is consistent with the concept of residual inflammatory and thrombotic risk, where Lp(a) may exacerbate the underlying atherothrombotic burden in already vulnerable patients. The GRACE score integrates multiple clinical variables, including age, heart rate, systolic blood pressure, creatinine, cardiac biomarkers, and ST-segment deviation, and is well-validated for risk stratification in ACS patients. The interaction between Lp(a) and GRACE score suggests that Lp(a) measurement may be particularly valuable in refining risk prediction among intermediate-risk patients, where it could help guide intensity of secondary prevention therapies. This finding aligns with a study by Zhang et al., which demonstrated that Lp(a) was predictive of long-term mortality in ACS patients undergoing PCI, but the predictive value was enhanced when combined with established risk scores [24].

The innovative approach by Saeki and colleagues, examining changes in Lp(a) over time rather than baseline levels alone, represents an important conceptual advance [12]. Their finding that a greater decrease in Lp(a) from baseline to 12 months was associated with lower MACE risk, while baseline Lp(a) alone was not predictive, suggests that dynamic changes in Lp(a) may offer superior prognostic information. This finding has several important implications. First, it suggests that a single Lp(a) measurement at the time of PCI may not fully capture an individual's risk trajectory, and that serial measurements could provide additional value. Second, it raises the possibility that interventions that lower Lp(a) over time, whether through lifestyle modifications, optimized medical therapy, or emerging Lp(a)-targeted agents, may translate into clinical benefit. Third, it highlights the importance of considering Lp(a) as a modifiable risk factor rather than a fixed genetic marker. While Lp(a) levels are primarily genetically determined, they can be influenced by oestrogen status, renal function, thyroid hormone, and emerging pharmacological agents, including PCSK9 inhibitors, antisense oligonucleotides, and small interfering RNA therapies targeting Lp(a) production. The ongoing phase 3 trials of pelacarsen and olpasiran will provide definitive evidence regarding whether Lp(a) lowering translates into cardiovascular event reduction, and the findings from Saeki and colleagues suggest that such benefits may be detectable within relatively short timeframes.

The association between elevated Lp(a) and stent-related complications, particularly stent thrombosis, is a clinically important finding with mechanistic implications. Her and colleagues reported a striking 83% increased risk of stent thrombosis with Lp(a) ≥50 mg/dL, while Wu and colleagues demonstrated increased risk of acute stent thrombosis with Lp(a) ≥30 mg/dL [10,17]. Stent thrombosis is a rare but devastating complication with high mortality, and identifying modifiable risk factors is of paramount importance. The prothrombotic effects of Lp(a) are mediated through several mechanisms: its structural homology with plasminogen allows it to compete for binding sites and inhibit fibrinolysis; it promotes platelet aggregation and activation; and it carries oxidised phospholipids that activate inflammatory pathways and tissue factor expression. These findings suggest that patients with elevated Lp(a) may require more intensive and prolonged antiplatelet therapy, although this hypothesis requires prospective evaluation. The association with repeat revascularisation, consistently observed across multiple studies, likely reflects accelerated progression of atherosclerosis in native vessels and possibly accelerated in-stent restenosis, although the latter mechanism is less well-established in the DES era [10,13,16].

The measurement heterogeneity observed across studies reflects the ongoing challenges in standardizing Lp(a) assays. Studies employed immunoturbidimetric assays, immunonephelometric assays, ELISA, and unspecified hospital assays, with variable reporting of whether results were reported in mass units (mg/dL) or molar units (nmol/L). This heterogeneity complicates direct comparison of effect sizes across studies and underscores the need for standardization in clinical practice and research. The International Federation of Clinical Chemistry and Laboratory Medicine has developed a secondary reference material for Lp(a) standardization, and contemporary assays are increasingly calibrated to this standard. However, many of the studies included in this review predate widespread adoption of standardised assays, and variability in Lp(a) measurements may have introduced non-differential misclassification, potentially biasing results toward the null. If anything, this measurement heterogeneity may have led to underestimation of the true association between elevated Lp(a) and outcomes, strengthening confidence in the positive findings observed.

The risk of bias assessment using the NOS demonstrated high methodological quality across most included studies, with seven of eight studies rated as high quality. Three studies achieved the maximum score of nine stars, reflecting robust selection methods, excellent control for confounding, and comprehensive outcome assessment with long-term follow-up and independent adjudication [10,15,16]. The consistency of findings across studies of varying design, geography, and methodological quality strengthens the evidence base and supports the robustness of the observed associations. The one study rated as moderate quality due to limited confounding control and restriction to in-hospital outcomes nevertheless contributed important data on acute stent thrombosis and in-hospital complications, domains not addressed by other studies [17].

Limitations

This systematic review has several limitations that should be considered when interpreting the findings. First, all included studies were observational in design, which introduces the potential for residual confounding despite multivariable adjustment in most studies. Unmeasured or incompletely measured confounders, such as inflammatory markers, genetic factors, or adherence to medications, may have influenced the observed associations. Second, significant heterogeneity existed across studies in terms of Lp(a) thresholds (ranging from ≥30 mg/dL to ≥50 mg/dL), outcome definitions, measurement methods (immunoturbidimetric, immunonephelometric, ELISA, and unspecified assays), and follow-up durations (from in-hospital to 7.4 years). This heterogeneity precluded meta-analysis and limits direct comparison of effect sizes across studies. Third, publication bias remains a concern, as studies with null or negative findings may be less likely to be published, although the inclusion of one study with null findings partially mitigates this concern. Fourth, the majority of studies were conducted in Asian populations (China, South Korea, Japan, India), which may limit generalizability to other ethnic groups, particularly given known ethnic variations in Lp(a) distribution. Fifth, several studies did not report whether Lp(a) measurements were performed using standardized assays calibrated to the International Federation of Clinical Chemistry and Laboratory Medicine reference material, which may have introduced measurement variability. Sixth, information on important covariates such as specific antiplatelet regimens, duration of dual antiplatelet therapy, and adherence to statin therapy was not consistently reported across studies, precluding assessment of potential interactions. Seventh, the restriction to English-language publications may have introduced language bias. Finally, the small number of included studies (n=8) limits the robustness of subgroup analyses and assessment of publication bias.

## Conclusions

Elevated Lp(a) is independently associated with an increased risk of MACEs, myocardial infarction, stent thrombosis, and repeat revascularization after PCI. The association is consistent across diverse populations and study designs, with hazard ratios ranging from 1.14 to 4.29 for MACE. The prognostic value of Lp(a) may be attenuated by aggressive LDL-C lowering and is most pronounced in high-risk patients and those with diabetes. Dynamic changes in Lp(a) over time may offer prognostic information beyond baseline measurements, suggesting the potential value of serial assessment. These findings may support consideration of Lp(a) measurement in patients undergoing PCI to improve risk stratification and guide the intensity of secondary prevention therapies, particularly LDL-C lowering, while acknowledging the limitations inherent to the observational nature of the included studies. The development of potent Lp(a)-lowering agents offers promise for directly targeting this residual risk factor, and the PCI population with elevated Lp(a) represents a high-risk group that could potentially benefit from such therapies. Future research should focus on standardizing Lp(a) assessment, elucidating the mechanisms linking Lp(a) to adverse outcomes after PCI, and evaluating the efficacy of emerging Lp(a)-lowering therapies in improving long-term prognosis in this population.
